# Implementing a digital audit-and-feedback surveillance system for diabetic ketoacidosis: study protocol for the DEKODE programme

**DOI:** 10.3389/frhs.2026.1836949

**Published:** 2026-09-09

**Authors:** Amanda Ling Jie Yee, Kalyaani Persad, Aspasia Manta, Amy Grove, Punith Kempegowda

**Affiliations:** 1 Queen Elizabeth Hospital Birmingham, University Hospitals Birmingham NHS Foundation Trust, Birmingham, United Kingdom; 2 Department of Applied Health Sciences, School of Health Sciences, College of Medicine and Health, University of Birmingham, Birmingham, United Kingdom; 3 Sandwell and West Birmingham NHS Foundation Trust, Birmingham, United Kingdom; 4 Centre for Evidence and Implementation Science, University of Birmingham, Birmingham, United Kingdom; 5 Department of Diabetes and Endocrinology, University Hospitals Birmingham NHS Foundation Trust, Birmingham, United Kingdom

**Keywords:** audit and feedback, DEKODE, diabetic ketoacidosis, digital health, implementation science, surveillance

## Abstract

**Introduction:**

Diabetic ketoacidosis is one of the leading causes of unplanned hospital admission among people with diabetes, yet adherence to treatment guidelines remains inconsistent, contributing to variation in care and suboptimal outcomes. Conventional audit approaches are limited by non-standardised data collection, restricting benchmarking and shared learning. The Digital Evaluation of Ketosis and Other Diabetic Emergencies (DEKODE) platform was developed as a cloud-based audit-and-feedback system to support near real-time performance monitoring and continuous quality improvement, but its implementation requires theory-informed evaluation.

**Methods:**

This multi-site, mixed-methods study involves six National Health Service hospitals, including early adopters and sites new to DEKODE. Guided by an implementation research logic model, the study links contextual determinants to tailored strategies. Quantitative outcomes, including episode duration, guideline adherence, complications, and clinical outcomes, will be analysed using interrupted time series methods. Qualitative stakeholder interviews will be analysed thematically, and findings integrated using triangulation.

**Results:**

The study will assess the impact of DEKODE on clinical and process outcomes and identify determinants influencing adoption, fidelity, and sustainability.

**Discussion:**

Findings will inform scalable implementation of digital audit-and-feedback systems to reduce variation in acute diabetes care and improve patient outcomes.

**Clinical Trial Registration:**

https://www.isrctn.com/ISRCTN17096714, identifier ISRCTN17096714.

## Introduction

Diabetic ketoacidosis (DKA) is one of the leading causes of unplanned hospital admissions among people with diabetes and remains a major contributor to avoidable morbidity, mortality, and prolonged hospitalisation (1). Audits and quality improvement (QI) initiatives have been conducted in individual hospitals to evaluate and enhance DKA care (2, 3). Despite the availability of clear, evidence-based protocols in local, national, and international guidelines, adherence to recommendations remains inconsistent (4, 5). This inconsistency highlights a fundamental implementation challenge: the problem is not a lack of knowledge, but rather ensuring that effective care is delivered reliably, consistently, and sustainably in routine clinical practice (6). In implementation science, this is referred to as a knowledge translation gap (7).

A limitation of current improvement efforts is the lack of standardised data collection methods across sites (8, 9). Without consistent, comparable data, it is difficult to identify and disseminate best practices, benchmark performance, or support cross-institutional learning (10). Although various interventions—such as protocol updates, targeted education, and intermittent performance feedback—have shown short-term gains (11), improvements are rarely sustained (12). This underscores the need to apply theory-informed implementation approaches to understand the contextual factors that influence uptake, the mechanisms driving change, and the strategies required to sustain improvements over time (13). Without such approaches, interventions risk being fragmented, non-transferable, and vulnerable to decline once initial efforts subside.

To address these challenges, we developed the Digital Evaluation of Ketosis and Other Diabetic Emergencies (DEKODE) platform—a cloud-based audit and feedback (A&F) model designed to embed continuous quality improvement into the delivery of DKA care (14). DEKODE standardises data collection across hospitals, enables near real-time and longitudinal monitoring of process and outcome measures, provides quarterly performance feedback, and benchmarks institutional performance internally and externally (11). The design of DEKODE is adaptable to diverse healthcare settings, and can be embedded within existing hospital workflows, facilitating timely access to performance data for clinical teams and leadership (14). DEKODE enables rapid identification of care gaps, targeted interventions (14), and interactive refinements—such as monitoring for iatrogenic complications (14), increasing feedback frequency (11, 15), and integrating outcome-linked quality metrics (14).

Implementation of DEKODE has been guided by clinical performance feedback intervention theory (16). It began when a group of consultants and resident doctors, motivated to improve diabetic care, came together to initiate change. Since then, the program has relied on regular feedback on the clinical performance of individual hospitals compared with their baseline performance. The approach incorporates components identified in a 2025 update of a Cochrane review (17) as yielding the greatest A&F effect sizes through meta-regression:

- providing data directly to individual hospitals rather than only at a group level
- comparing institutional performance against benchmarks
- using interactive methods—such as regular meetings—rather than solely didactic or written formats to deliver feedback
- applying facilitation strategies to support engagement
- developing action plans through agreements with local consultants to drive practice change within hospitals

The DEKODE platform has also been successfully applied to other diabetic emergencies, including hyperosmolar hyperglycaemic state (HHS) and severe hypoglycaemia, demonstrating its adaptability and broader potential within acute diabetes care. These applications provide practical evidence that the model can be extended beyond DKA to other high-risk, time-sensitive conditions, supporting both local quality improvement and system-wide learning. Although informed by practical experience, DEKODE has not yet been rigorously evaluated within an implementation science framework.

This paper describes the design, implementation, and evaluation framework underpinning DEKODE, positioning it as a potentially scalable digital intervention for improving DKA care. The specific objectives for this implementation study are:

1. To identify and examine the facilitators and barriers to the set up, sustainability, and scalability of DEKODE using an evidence-informed implementation science framework.
2. To evaluate how DEKODE contributes to improvements in DKA outcomes among existing and newly participating hospitals.

Building on prior publications (14, 18) that have demonstrated DEKODE's utility, we now seek to situate it within an implementation science framework, specifically the Implementation Research Logic Model (IRLM). This work addresses an important gap in the literature by offering a theory-informed understanding of how improvements in DKA care can be achieved and sustained, while providing practical insights for scaling, adaptation, and replication across other acute care pathways.

## Methods

### Study design and setting

This multi-site, mixed-methods implementation study will be conducted within the Department of Applied Health Sciences at the University of Birmingham, with ethical approval obtained from the Health Research Authority (Ref: 25/IEC08/0011). The study protocol was registered with the ISRCTN registry (ISRCTN17096714; https://www.isrctn.com/ISRCTN17096714). The study will run from March 2026 to June 2028, preceded by a recruitment and onboarding phase starting in November 2025. Reporting of the study will adhere to the Standards for Reporting Implementation Studies (STaRI) checklist to ensure transparency, methodological rigour, and consistency in the dissemination of implementation research findings (Supplementary Material S1) (19).

### Patient and public involvement

Patient and public involvement activities were undertaken from the proposal design stage and will continue to inform dissemination plans. We consulted public members of the Joint British Diabetes Societies (JBDS) Patient Panel, the Diabetes UK Communities in Action group, and people with diabetes from the West Midlands region. These contributors also have links with national and international diabetes organisations, supporting the inclusion of wider patient perspectives relevant to diabetes care and policy.

The purpose of the consultation was to elicit the views of people with diabetes on the value of the study and to explore safety and ethical considerations associated with the proposed methods, particularly for work packages involving patients, carers, and healthcare professionals. Following an introductory discussion, a 30-minute Zoom meeting was held with two members of Diabetes UK Communities in Action. Feedback from this consultation supported the relevance of the study and informed the study design, qualitative interview approach, implementation evaluation, language refinement, and dissemination plans.

### Implementation science framework

Earlier evaluations demonstrated DEKODE's feasibility and potential to improve DKA outcomes (14); However, uptake has varied considerably across hospitals, with some sites integrating DEKODE into routine workflows more rapidly than others. A key limitation has been inconsistent clinician engagement (20, 21), with some perceiving performance feedback as a threat to professional identity rather than an opportunity for improvement (22). Such challenges underscore the importance of addressing the relational and cultural dimensions of implementation alongside technical aspects. We recognise that knowledge context–strategy–mechanism relationships, as articulated in the IRLM framework, will be central to supporting sustainability and scalability in the next phase of work (23).

To address the existing variations, we have chosen the IRLM as the overarching framework for the study's design, data collection, analysis, and interpretation (24).

Implementation research often draws on multiple frameworks to address determinants, processes, and evaluation, but integrating this led to fragmented strategies and conceptual ambiguity (25). The IRLM mitigates this challenge by providing a single, integrative structure that explicitly links contextual determinants, implementation strategies, mechanisms of action, and both implementation and clinical outcomes (25).

IRLM enables the consolidation of complementary frameworks–such as the Consolidated Framework for Implementation Research (CFIR), Reach, Effectiveness, Adoption, Implementation, and Maintenance (RE-AIM), and Theoretical Domains Framework (TDF) while maintaining conceptual clarity (24), rather than using each framework individually, which can result in omission of important information. It has been successfully applied across various healthcare settings (26–28). A national evaluation of IRLM utility found that over 50% of respondents rated it as “moderately” or “very helpful” for usability, conceptual clarity, and applicability to real-world challenges (29). Its graphic depiction further facilitates transparency, stakeholder communication, and reproducibility, which are valuable for multisite digital interventions such as DEKODE (24).

**Figure 1:**
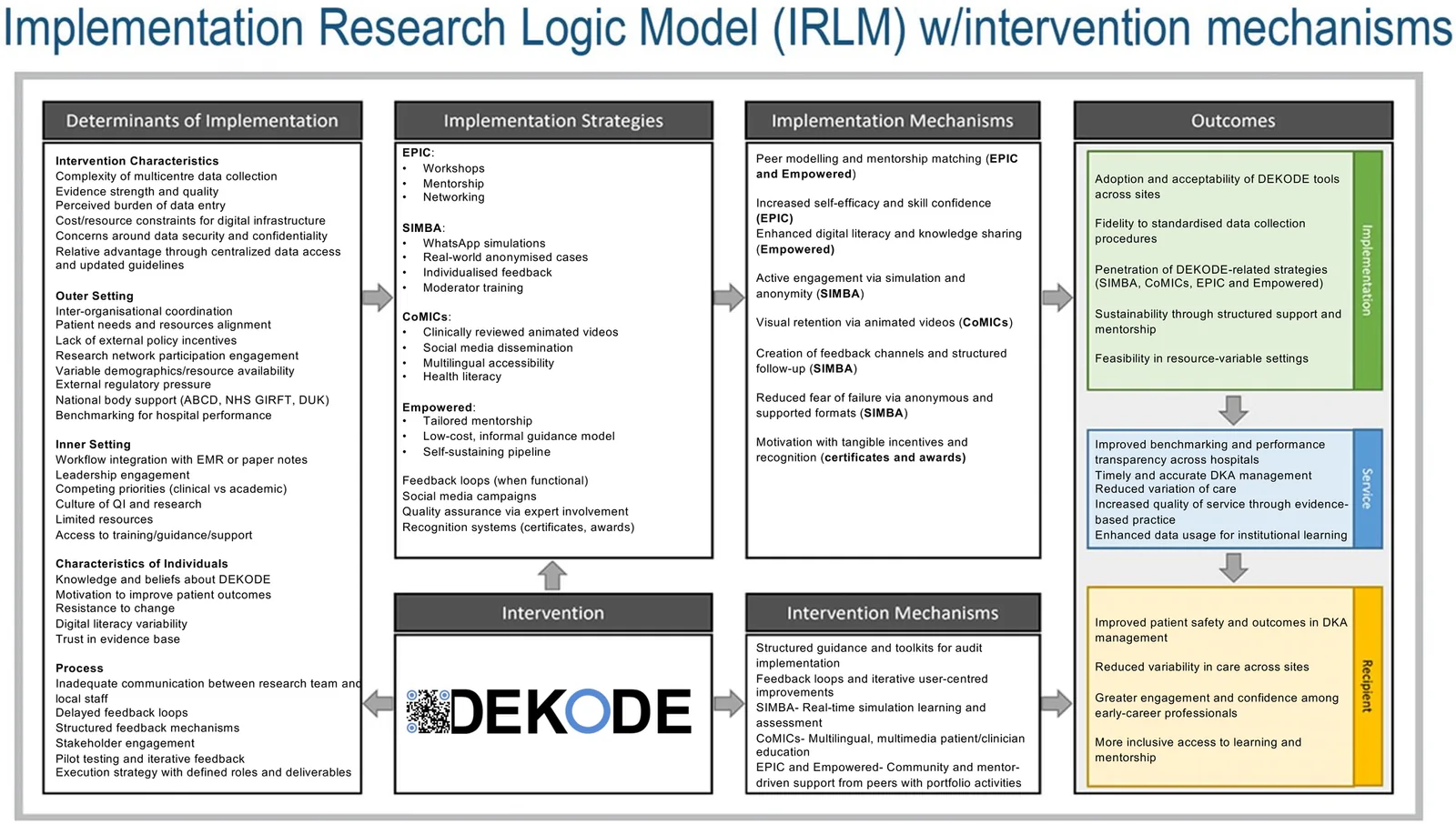
Adapted IRLM for the DEKODE initiative. The IRLM illustrates the overarching structure linking implementation determinants, strategies, mechanisms, and outcomes underpinning the DEKODE implementation approach. Determinants were identified using the CFIR and mapped across the five IRLM domains: Intervention Characteristics, Outer Setting, Inner Setting, Characteristics of Individuals, and Process. Implementation strategies were selected and operationalised using the ERIC taxonomy to address identified barriers and leverage facilitators. These strategies are hypothesised to act through mechanisms such as increasing knowledge, engagement, confidence, peer learning, and organisational support, leading to improved implementation outcomes (adoption, fidelity, penetration, sustainability, feasibility) and ultimately improved clinical outcomes in DKA care.

The IRLM serves as the overarching framework for this study by providing a structured representation of the relationships between implementation determinants, strategies, mechanisms, and outcomes. Within the IRLM, the CFIR is used to identify and categorise contextual determinants that may influence DEKODE implementation, including factors related to the intervention itself, organisational context, individual users, and implementation processes. These determinants are then linked to implementation strategies using the Expert Recommendations for Implementing Change (ERIC) taxonomy (30), which provides a comprehensive set of evidence-informed strategies to address identified barriers and strengthen facilitators. CFIR informs what implementation challenges exist, ERIC informs how those challenges may be addressed, and the IRLM integrates these relationships into a coherent causal pathway linking DEKODE implementation activities to mechanisms of changes, implementation outcomes, and clinical outcomes. We have hypothesised mechanisms of action through which selected strategies are expected to enhance fidelity, adoption, and sustainability, ultimately leading to improved patient outcomes through the improvement management of DKA. This information is mapped in our adapted IRLM (Figure 1).

### Site selection and sample size rationale

We will purposely select six National Health Service (NHS) hospitals across England to participate in the study. All centres who have been using the DEKODE platform for at least twelve months will be invited to participate in this research. From this, three centres who are interested to participate in the study following informed consent will be included on a first come basis. Similar, all new interested in joining the DEKODE surveillance system will be invited to participate in the study as well. The three centres interested to participate after informed consent will be included in the study on a first come basis as well. This sampling strategy is designed to capture implementation processes across sites with varying levels of experience, organisational structures, resources, and clinical workflows. This approach balances practical feasibility of delivering the evaluation with the need to explore variability, enhancing the transferability of findings to broader NHS settings.

The number of participating centres was selected pragmatically to balance feasibility with the need to capture variation in organisational context, clinical workflow, and implementation maturity. The inclusion of three early adopter sites is informed by previous DEKODE work, in which data from 177 DKA episodes across three hospitals were used to evaluate variation in DKA care processes, examine factors influencing length of stay and test quality improvement interventions. Although that study did not demonstrate a significant reduction in. DKA duration or length of stay, it demonstrated the feasibility of using a three-hospital DEKODE dataset to support multi-side audit, benchmarking, and quality improvement. The present study expands this approach by including three early adopter sites and three new implementer sites, enabling comparison across different levels of implementation maturity and hospital context. For the quantitative component, all eligible adult DKA episodes identified during the baseline, implementation, and follow-up periods will be included, with the final number determined by site-level DKA admission volumes.

### Eligibility criteria and case definition

Eligible episodes will include adults aged ≥16 years who are admitted with DKA or who develop DKA during hospital admission at a participating centre during the study period. All types of diabetes will be eligible, provided that the episode meets the diagnostic criteria for DKA according to the Joint British Diabetes Societies (JBDS) DKA guideline.

In line with the JBDS DKA guideline, DKA will be defined by the presence of hyperglycaemia or known diabetes, ketonaemia or significant ketonuria, and metabolic acidosis. This will generally include capillary or blood ketones ≥3.0 mmol/L or significant ketonuria, venous or arterial pH <7.3 and/ or serum bicarbonate <15 mmol/L, alongside clinical assessment consistent with DKA.

Each DKA presentation will be treated as a separate episode for audit and feedback purposes. Recurrent DKA admissions in the same individual will therefore be included as separate episodes, as each presentation represents a distinct episode of care and an opportunity to evaluate DKA management.

Episodes will be excluded if the JBDS diagnostic criteria for DKA are not met, if the presentation represents isolated ketosis without acidosis, hyperosmolar hyperglycaemia state without DKA, or if essential data required to confirm DKA diagnosis are unavailable.

### DEKODE implementation process

Interested participating hospitals will be provided with access to an online study folder containing the study protocol and data collection form, which will support local audit registration. Following local registration, each site will share its audit registration number with the central DEKODE team and attend a virtual onboarding meeting covering the study protocol, eligibility criteria, and data collection procedures.

Each participating site will identify a local centre lead, who will coordinate communication between the local team and the central DEKODE team. Data collection will primarily be undertaken by clinicians and medical students involved in the DEKODE project, following audit registration and training. Local audit, governance, and quality improvement structures will be engaged to support approval, oversight, and alignment of the initiative with existing hospital processes.

Implementation will involve site onboarding, local audit registration, training of local data collectors and clinical leads, standardised data collection through REDCap-based DEKODE platform, quarterly audit-and-feedback reports, and structured local action planning. Each hospital will obtain a patient list from its information team for adults aged ≥16 years who were admitted with DKA or developed DKA during admission. Patient-identifiable data will remain within the local hospital team and will not be shared with the central DEODE team. The central team will receive only pseudonymised data through REDCap.

Standardisation across participating sites will be supported through use of common study protocol, eligibility criteria, data collection form, data dictionary, and REDCap-based data submission platform. All sites will collect the same core dataset for each eligible DKA episode, including diagnostic criteria, key timestamps, biochemical monitoring, treatment processes, complications, and clinical outcomes. The virtual onboarding meeting and pilot review of five DKA episodes will be used to ensure that local data collectors interpret variables consistently before full data collection begins. This approach is intended to support comparable data collection, benchmarking, and cross-site learning while allowing local adaptation in how improvement actions are implemented.

Although data collection will be led by the local DEKODE team, the quarterly feedback reports may identify care gaps that require wider multidisciplinary input. Depending on local findings, improvement actions may involve diabetes and endocrinology clinicians, acute medicine and emergency department teams, nursing staff, pharmacy, dietetics or nutrition teams, information or digital teams, patient safety teams, and operational leadership. This approach ensures that DEKODE remains operationally feasible as a clinician-led audit-and-feedback programme, while allowing wider multidisciplinary engagement when specific improvement interventions require it.

To ensure data quality, each site will initially submit five pilot DKA episodes for central review. The DEKODE team will assess data completeness, accuracy and consistency, and provide feedback before full data collection continues for the agreed study period. Submitted data will then be monitored for completeness, consistency and plausibility, with queries returned to the local centre lead for clarification or correction using local source records where required.

Participating hospitals will receive feedback reports every three months, summarising local processes and outcome measures. Local teams will use these reports to identify gaps in DKA care and decide on appropriate context-specific improvement interventions. In addition, monthly meetings will be held with all participating centres to discuss progress, share learning, address challenges, and support consistency across sites. A virtual group communication channel will also be established to enable real-time communication between participating centres and the central DEKODE team. This will allow implementation issues, data collection queries, and site-specific challenges to be identified promptly and addressed iteratively.

Implementation fidelity will be monitored by assessing whether core DEKODE components are delivered as intended, including local audit registration, completion of onboarding, submission of pseudonymised data, pilot data review, quarterly feedback reporting, and participation in ongoing communication or feedback processes. Local adaptations and implementation challenges will be documented to distinguish necessary contextual tailoring from deviation from core intervention components. These implementation tools and strategies will operationalise the IRLM, CFIR, and ERIC-informed approach by linking identified contextual barriers to practical actions, feedback mechanisms, local adaptations, and follow-up improvement planning at each participating site.

### Quantitative data collection and outcomes

The study will include a six-month baseline period prior to DEKODE implementation or evaluation (January to June 2026), a six-month active implementation phase (July to December 2026), and a twelve-month follow-up period (January to December 2027). This proposed timeline is informed by previous DEKODE evaluations, in which improvements in DKA care episode duration were detectable within a comparable follow-up period after implementation (11). An interrupted time series (ITS) design will be applied to assess changes in outcomes attributable to DEKODE implementation.

The primary quantitative outcomes include the DKA episode duration, defined as the time from diagnosis to biochemical resolution; adherence to the Joint British Diabetes Societies (JDBS) guidelines for DKA between 80% and 120% of the recommendations, such as timely initiation of insulin treatment, adequate fluid resuscitation, and appropriate biochemical monitoring; and the occurrence of iatrogenic complications, including hypokalaemia and hypoglycaemia. In addition, we will assess key clinical outcomes, namely in-hospital mortality, 30-day readmission rates for DKA, and overall length of hospital stay. Secondary outcomes will focus on comparative performance between early adopter and new implementer sites, as well as site-level variation relative to the collective dataset, enabling benchmarking across participating hospitals.

### Quality improvement and implementation measures

The practical improvement component of DEKODE will be informed by concepts from the Model of Improvement (31). The specific aims are to reduce unwarranted variation in DKA care across participating centres and to understand how DEKODE is implemented, adapted, and sustained in different hospital contexts.

These aims will be addressed through mixed-methods approach. Quantitative audit data will be used to examine DKA process and clinical outcomes. Process measures will include adherence to key components of DKA management, including timely initiation of insulin, appropriate fluid replacement, ketone monitoring, glucose monitoring, and potassium monitoring. Clinical outcome measures will include time to DKA resolution, length of hospital stay, hypoglycaemia, hypokalaemia, intensive care admission, 30-day readmission, and mortality.

Qualitative data from healthcare professionals involved in DKA care, patients recently hospitalised with DKA and carers or family members where appropriate will be used to explore perspectives on DKA management and implementation experiences. This will include perceived adoption, fidelity, feasibility, acceptability, local adaptations, barriers, and facilitators to DEKODE implementation and sustained use.

Balancing indicators will include unintended consequences of implementation, such as additional data collection burden, variable clinician engagement with feedback and potential impact on local workload. Implemented changes will be monitored through quarterly feedback reports, monthly cross-site meetings, and real-time communication between participating centres and the central DEKDOE team. These mechanisms will allow local teams to review progress, identify barriers, refine improvement actions, and adapt implementation strategies according to local context.

### Missing data

The extent and pattern of missing data will be summarised by variable, site, and study phase. For quantitative analyses, cases will be included where the required data for a specific outcome are available. Therefore, an episode with missing data for one secondary outcome may still contribute to analyses of other outcomes where the relevant data are complete. Episodes missing essential data required to confirm DKA diagnosis or calculate the primary outcome will be excluded from the relevant analysis. Where missingness is substantial or differs across sites or study phases, this will be reported and considered when interpreting the findings.

#### Survey and qualitative interviews

In addition to collecting clinical outcome data, we will administer a structured survey to assess stakeholder perceptions of the determinants and strategies influencing DEKODE uptake. Candidate determinants will be identified from prior DEKODE studies, relevant CFIR constructs, and rapid consultation with external stakeholders. Participants (clinicians and managers involved in DKA care at each site) will be asked to rate the importance of each determinant on a 5-point Likert scale ranging from 1 (not important) to 5 (extremely important). An open-text option will also be provided to allow respondents to suggest additional factors or strategies not listed. Quantitative survey data will be analysed descriptively, whilst the open-text responses will undergo thematic coding and be integrated with qualitative interview findings.

Semi-structured interviews will be conducted with participants at each site, focusing on exploring determinants, strategies, mechanisms, and perceived outcomes of DEKODE implementation. Interview participants will include healthcare professionals directly involved in DKA care, patients recently hospitalised with DKA along with their family members, and hospital managers or digital transformation lead.

For the qualitative component, we aim to recruit approximately 8–13 participants per site (32). This is expected to generate approximately 66 interviews across all participating sites. This sample is anticipated to provide sufficient breadth across stakeholder groups and implementation contexts to achieve data saturation (33). Saturation will be assessed iteratively during analysis, based on the recurrence of key themes, codebook stability, and the absence of substantially new concepts in later interviews. Patients recently hospitalised with DKA and carers or family members where appropriate will be approached post-discharge by clinical teams to minimise burden, with ethical safeguards including a distress protocol and support signposting.

Interviews will take place twice during the study, each involving a distinct cohort of participants: first, within six to nine months after DEKODE activation to capture initial implementation experiences, and later near the end of the study period to assess sustained engagement, perceived benefits, and any ongoing barriers or facilitators. The interview guides will be piloted and refined at an initial site before use across all participating centres. All interviews will be audio-recorded, transcribed verbatim, and pseudoanonymised to protect participant confidentiality.

### Qualitative analysis

The qualitative data will be analysed using deductive-inductive hybrid thematic approach (34). Analysis will begin with deductive coding guided by the five domains of the CFIR: intervention characteristics, inner setting, outer setting, characteristics of individuals, and process. This will be followed by inductive coding to identify emerging themes not captured by CFIR. Retroduction is then adopted to theorise underlying generative mechanisms that explain observed patterns across sites. Coding and analysis will be supported by NVivo software. Findings will be mapped back to the IRLM, ensuring that determinants, strategies, and mechanisms are explicitly linked to outcomes.

### Mixed-methods integration

To integrate the qualitative and quantitative findings, we will employ a triangulation protocol (35) using the IRLM (24) as the organising framework. We will systematically identify convergence, complementarity, dissonance, and silence across data sources. This process will synthesise insights (36), enabling us to link contextual determinants, implementation strategies, mechanisms, and outcomes within a unified analytic structure. Triangulation will help us uncover patterns that inform targeted refinements and guide the effective scaling of DEKODE across diverse NHS settings.

## Discussion

This study contributes to implementation science by offering a detailed, theory-informed method for embedding a scalable digital audit and feedback system within routine acute care pathways. By explicitly integrating established frameworks such as IRLM, the method provides a structured approach to identify the contextual barriers and facilitators that influence DKA care, systematically link these facilitators and barriers to targeted implementation strategies, and map the mechanisms through which these strategies improve both process and clinical outcomes. Our approach ensures that DEKODE is not applied in isolation but is tailored to local workflows, resource availability, and organisational culture. Unlike approaches that focus solely on clinical effectiveness or isolated quality improvement projects (37, 38), this framework-driven design allows DEKODE to address the root causes of variability in DKA care, enabling consistent, scalable improvements across diverse hospital settings. Applied to DKA care, DEKODE is expected to reduce variation in practice, enhance protocol adherence, and thereby indirectly improve patient outcomes across diverse hospital settings.

For researchers, our study provides a replicable model to evaluate digital interventions in healthcare, emphasising mixed methods approaches and rigorous framework application. Practitioners may benefit from actionable insights into how data-driven feedback can be integrated to enhance clinical workflows and patient outcomes (14, 18).

A key implementation challenge will be translating the DEKODE model from a theory-informed digital audit-and-feedback intervention into routine clinical practice across hospitals with different levels of organisational maturity, staffing capacity, digital infrastructure, and safety culture. Although standardised data collection and feedback reports provide a common structure, sustained improvement is likely to depend on local ownership, multidisciplinary engagement, and the ability of teams to act on feedback within existing governance and quality improvement structures. Sites may vary in their readiness to engage with performance feedback, their capacity to complete data collection, and their ability to implement improvement actions in response to identified care gaps. These factors may influence both the fidelity and sustainability of DEKODE implementation.

The study will therefore examine not only whether DEKODE is associated with changes in DKA care but also how local context shapes implementation. Understanding barriers such as workload pressures, limited protected time, variable engagement the feedback, and concerns about benchmarking will be important for interpreting differences between sites. Similarly, identifying facilitators such as clinical leadership, psychological safety, multidisciplinary collaboration, and alignment with existing audit or patient safety priorities may help explain why some centres embed DEKODE more successfully than others. These insights will be important for refining the intervention and informing future scale-up across acute diabetes and wider emergency care pathways.

Following completion of this study, future research could explore the application of this digital audit-and-feedback surveillance system in other acute and chronic care settings, investigate long-term sustainability beyond initial implementation phases, and refine strategies to optimise engagement in resource-limited environments. Continued refinement of digital platforms like DEKODE, informed by iterative evaluation and evolving health system needs, will be essential to maximising their impact on healthcare environments, clinical practices, and patient needs are constantly changing, and ongoing adaptation ensures the platform remains relevant, effective, and capable of improving outcomes.

### Strengths and limitations

The application of the IRLM in this study provides distinct advantages for evaluating DEKODE beyond generic framework use. First, it allows us to systematically capture why some hospitals rapidly integrate DEKODE into routine DKA workflows while others face resistance, by explicitly linking determinants such as staffing capacity, data governance, and perceptions of performance feedback to tailored implementation strategies. Second, it helps explain *how* audit-and-feedback mechanisms—such as benchmarking institutional performance, consultant-led facilitation, and interactive feedback meetings—translate into observable outcomes including shorter episode duration and improved adherence to JBDS protocols. Third, the IRLM offers a structure to balance fidelity with necessary adaptation, distinguishing between core DEKODE functions (e.g., real-time data capture, standardised reporting across sites) and local tailoring (e.g., integration with variable EHR systems). Finally, by aligning mixed-methods data within the IRLM, we can triangulate outcome trends with qualitative accounts of clinician engagement, concerns about professional identity, and patient perspectives. This provides not only a rigorous evaluation of whether DEKODE works, but also a transparent explanation of how it works across diverse NHS contexts, strengthening the evidence base for wider scalability to other diabetic emergencies.

However, some limitations warrant consideration. The purposive selection of sites, while ensuring diversity in readiness and context, may limit generalisability beyond comparable NHS settings. Reliance on self-reported and locally collected data introduces potential inaccuracies, despite fidelity monitoring. Variations in electronic health record systems, staffing, and local priorities may affect both data quality and the consistency of implementation. The 12-month follow-up period is sufficient to detect early change but may not capture long-term sustainability. Intervention effects observed early may not persist over time, implementation strategies may require longer to become fully embedded, and without extended follow-up, it is unclear whether improvements are maintained or fade, limiting insights into real-world, lasting impact.

Overall, this study is positioned to deliver both practical and theoretical insights into scaling a digital quality improvement intervention for DKA care. While acknowledging constraints around generalisability, data quality, and follow-up, the combination of a robust conceptual framework, multi-site design, and integrated mixed-methods approach provides a strong basis for generating actionable evidence to guide broader dissemination of DEKODE.

## Conclusion

DEKODE is an cloud-based A&F model which leverages IRLM to identify and address the contextual factors that facilitate or hinder adoption, sustainability, and scale-up of the platform. By systematically linking these facilitators and barriers to targeted implementation strategies, this platform may help to foster consistent adoption and long-term integration into routine care. Through real-time data monitoring and iterative feedback, DEKODE may enable measurable improvements in DKA outcomes across both experienced and newly adopting hospitals.

- This study represents the first implementation of an audit-and-feedback system targeting a diabetes-related condition, an area of critical importance given the high morbidity and mortality associated with diabetes.
- The intervention was implemented across a wide spectrum of healthcare professionals, from medical students to senior consultants, demonstrating its adaptability and potential for integration across levels of clinical training and service delivery.
- This work expands the limited evidence base on the application of the Implementation Research Logic Model within NHS and wider UK healthcare contexts, offering practical insights into its utility for complex service evaluations.
