# Supplementary material for: Implementing a digital audit-and-feedback surveillance system for diabetic ketoacidosis: study protocol for the DEKODE programme
Source: Front Health Serv. 2026 Sep 9;6:1836949. doi: 10.3389/frhs.2026.1836949 (PMC13597787; doi:10.3389/frhs.2026.1836949)
Supplement: Supplementary file 1 [file Datasheet1.pdf]

Implementation Research Logic Model (IRLM) w/intervention mechanisms

Determinants of Implementation

Intervention Characteristics
Complexity of multicentre data collection
Evidence strength and quality
Perceived burden of data entry
Cost/resource constraints for digital infrastructure
Concerns around data security and confidentiality
Relative advantage through centralized data access and updated guidelines

Outer Setting
Inter-organisational coordination
Patient needs and resources alignment
Lack of external policy incentives
Research network participation engagement
Variable demographics/resource availability
External regulatory pressure
National body support (ABCD, NHS GIRFT, DUK)
Benchmarking for hospital performance

Inner Setting
Workflow integration with EMR or paper notes
Leadership engagement
Competing priorities (clinical vs academic)
Culture of QI and research
Limited resources
Access to training/guidance/support

Characteristics of Individuals
Knowledge and beliefs about DEKODE
Motivation to improve patient outcomes
Resistance to change
Digital literacy variability
Trust in evidence base

Process
Inadequate communication between research team and local staff
Delayed feedback loops
Structured feedback mechanisms
Stakeholder engagement
Pilot testing and iterative feedback
Execution strategy with defined roles and deliverables

Implementation Strategies

EPIC:
- Workshops
- Mentorship
- Networking

SIMBA:
- WhatsApp simulations
- Real-world anonymised cases
- Individualised feedback
- Moderator training

CoMICs:
- Clinically reviewed animated videos
- Social media dissemination
- Multilingual accessibility
- Health literacy

Empowered:
- Tailored mentorship
- Low-cost, informal guidance model
- Self-sustaining pipeline

Feedback loops (when functional)
Social media campaigns
Quality assurance via expert involvement
Recognition systems (certificates, awards)

Intervention

DEKODE

Implementation Mechanisms

Peer modelling and mentorship matching (**EPIC and Empowered**)

Increased self-efficacy and skill confidence **(EPIC)**
Enhanced digital literacy and knowledge sharing (**Empowered**)

Active engagement via simulation and anonymity (**SIMBA**)

Visual retention via animated videos (**CoMICs**)

Creation of feedback channels and structured follow-up (**SIMBA**)

Reduced fear of failure via anonymous and supported formats (**SIMBA**)

Motivation with tangible incentives and recognition (**certificates and awards)**

Intervention Mechanisms

Structured guidance and toolkits for audit implementation
Feedback loops and iterative user-centred improvements
SIMBA- Real-time simulation learning and assessment
CoMICs- Multilingual, multimedia patient/clinician education
EPIC and Empowered- Community and mentor-driven support from peers with portfolio activities

Outcomes

Implementation

Adoption and acceptability of DEKODE tools across sites

Fidelity to standardised data collection procedures

Penetration of DEKODE-related strategies (SIMBA, CoMICs, EPIC and Empowered)

Sustainability through structured support and mentorship

Feasibility in resource-variable settings

Service

Improved benchmarking and performance transparency across hospitals
Timely and accurate DKA management
Reduced variation of care
Increased quality of service through evidence-based practice
Enhanced data usage for institutional learning

Recipient

Improved patient safety and outcomes in DKA management

Reduced variability in care across sites

Greater engagement and confidence among early-career professionals

More inclusive access to learning and mentorship
